# The Structure of *Rd*DddP from *Roseobacter denitrificans* Reveals That DMSP Lyases in the DddP-Family Are Metalloenzymes

**DOI:** 10.1371/journal.pone.0103128

**Published:** 2014-07-23

**Authors:** Jan-Hendrik Hehemann, Adrienne Law, Lars Redecke, Alisdair B. Boraston

**Affiliations:** 1 Department of Biochemistry & Microbiology, University of Victoria, Victoria, British Columbia, Canada; 2 Joint Laboratory for Structural Biology of Infection and Inflammation of the Universities of Hamburg and Lübeck, c/o DESY, Hamburg, Germany; CINVESTAV-IPN, Mexico

## Abstract

Marine microbes degrade dimethylsulfoniopropionate (DMSP), which is produced in large quantities by marine algae and plants, with DMSP lyases into acrylate and the gas dimethyl sulfide (DMS). Approximately 10% of the DMS vents from the sea into the atmosphere and this emission returns sulfur, which arrives in the sea through rivers and runoff, back to terrestrial systems via clouds and rain. Despite their key role in this sulfur cycle DMSP lyases are poorly understood at the molecular level. Here we report the first X-ray crystal structure of the putative DMSP lyase *Rd*DddP from *Roseobacter denitrificans*, which belongs to the abundant DddP family. This structure, determined to 2.15 Å resolution, shows that *Rd*DddP is a homodimeric metalloprotein with a binuclear center of two metal ions located 2.7 Å apart in the active site of the enzyme. Consistent with the crystallographic data, inductively coupled plasma mass spectrometry (ICP-MS) and total reflection X-ray fluorescence (TRXF) revealed the bound metal species to be primarily iron. A 3D structure guided analysis of environmental DddP lyase sequences elucidated the critical residues for metal binding are invariant, suggesting all proteins in the DddP family are metalloenzymes.

## Introduction

**Figure 1:**
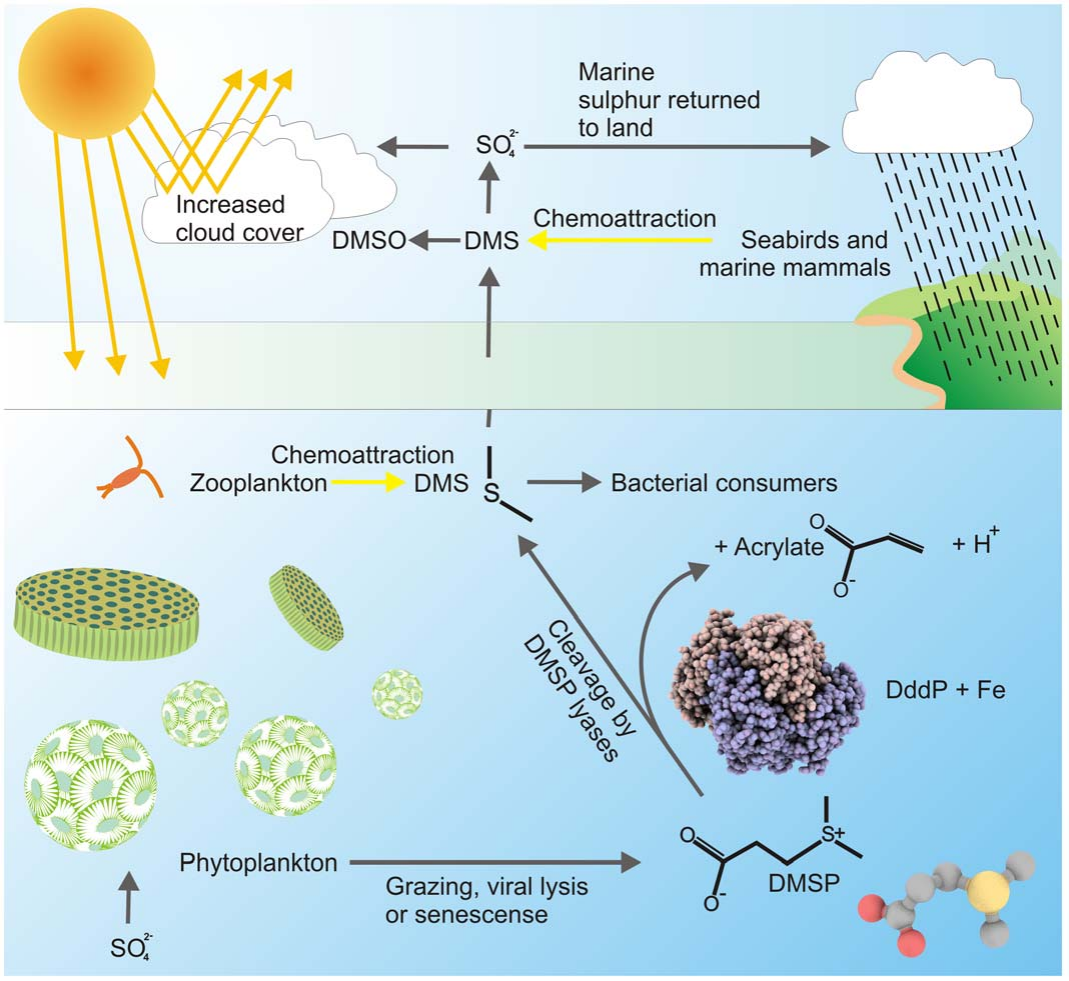
Microalgae produce DMSP; microbial lyases cleave DMSP into DMS, a process with relevant biogeochemical ramifications. (Adapted from [1]). A complete depiction of catabolic processing of DMSP by microbes is detailed in [6].

Dimethylsulfoniopropionate (DMSP) is a metabolite synthesized by marine algae and plants, (∼10^9^ tonnes per year [1]) where it functions as an osmolyte, chemical attractant-deterrent and possibly as an antioxidant precursor [2], [3], [4]. Blooms of single celled phytoplankton produce the bulk of DMSP (Figure 1), reaching concentrations of several µM in the surrounding seawater and intracellular concentrations of up to 1 M [5], [4].

Marine microbes are key in DMSP cycling [6] by catalyzing its cleavage into acrylate and dimethyl sulfide (DMS), thereby releasing ∼300 million tonnes of DMS per year [1]. Approximately 10% of the DMS vents from ocean waters and constitutes the major natural emission of sulphur to the atmosphere [4], returning the element in the form of oxidised DMS compounds within clouds and rain back to terrestrial systems (Figure 1), [7]. These sulfur compounds act as condensation nuclei, trigger cloud formation, increase the reflection of solar radiation and regulate climate [8]_,_ [9]. Therefore, microbial production of DMS is of broad significance for the sulfur cycle and climate. However, DMSP lyases are poorly understood at the molecular level.

Proteins in the DddP family are abundant and widely distributed in marine systems [10]. The first biochemically characterized DddP protein, which was cloned from *Roseovarius nubinhibens* ISM, displayed activity on DMSP to produce DMS and acrylate, albeit with relatively low activity under the assay conditions [11]. Furthermore, a mutant of *R. nubinhibens* lacking the gene encoding *Rn*DddP reduced the rate of DMS production by this marine bacterium by a factor of ∼8 and a transfer of this gene into *E. coli* conferred the ability to make DMS from DMSP [12]. Solidifying the identity of DddP proteins as DMSP lyases was the additional observation that the transfer of two fungal DddP enzymes, one from *Fusarium graminearum* cc19 and the other from *Aspergillus oryzae* RIB40 [12], into *E. coli* also conferred the ability to produce DMS from DMSP.

The DddP family of DMSP lyases is distantly related to the M24 family of metalloproteinases. These enzymes contain an active site with a binuclear metal center, which is crucial for their activity [13]. The residues that coordinate the metal cofactors in M24 metalloproteinases are also conserved in DddP lyases [12]. Initial reports characterizing the recombinant *Rn*DddP suggested the lack of metal cofactors; however, this was contradicted by the observation that when the residues conserved with M24 metalloproteinases were mutated to alanine in *Rn*DddP its DMSP lyase activity was abolished, suggesting these residues have an important biological function i.e. for metal binding and catalysis [11], [1].

To provide new insight into the molecular basis of DMSP cleavage by DddP enzymes we aimed to analyze a member of the DddP family using X-ray crystallography. We were able to crystallize and solve the structure of a putative DMSP lyase from the DddP family, *Rd*DddP from the marine bacterium *R. denitrificans* Och 114. This putative DMSP lyase shares 77% identity with *Rn*DddP, 50% identity with *Ao*DddP (*Aspergillus oryzae* RIB40) and 32% identity with *Fg*DddP (*Fusarium graminearum* cc19); these enzymes, including *Rd*DddP, are all part of the monophyletic clade that contains all three of the enzymes with confirmed functions [12]. Total reflection X-ray fluorescence (TRXF) and inductively coupled plasma mass spectrometry (ICP-MS) both revealed iron ions in *Rd*DddP. X-ray crystallography of *Rd*DddP revealed a binuclear metal center bound by the residues that are conserved with the metal binding residues in M24 metalloproteinases. Comparison with the amino acid sequences of the putative and the characterized DddP lyases showed these metal binding residues are invariant throughout the DddP family, and therefore likely critical for the biological function of these enzymes [11]. Together, these results provide strong evidence that DddP lyases are metalloenzymes and that iron may be of relevance to the cycling of DMSP by marine microbes.

## Results

### Presence of metal ions in *Rd*DddP

**Figure 2:**
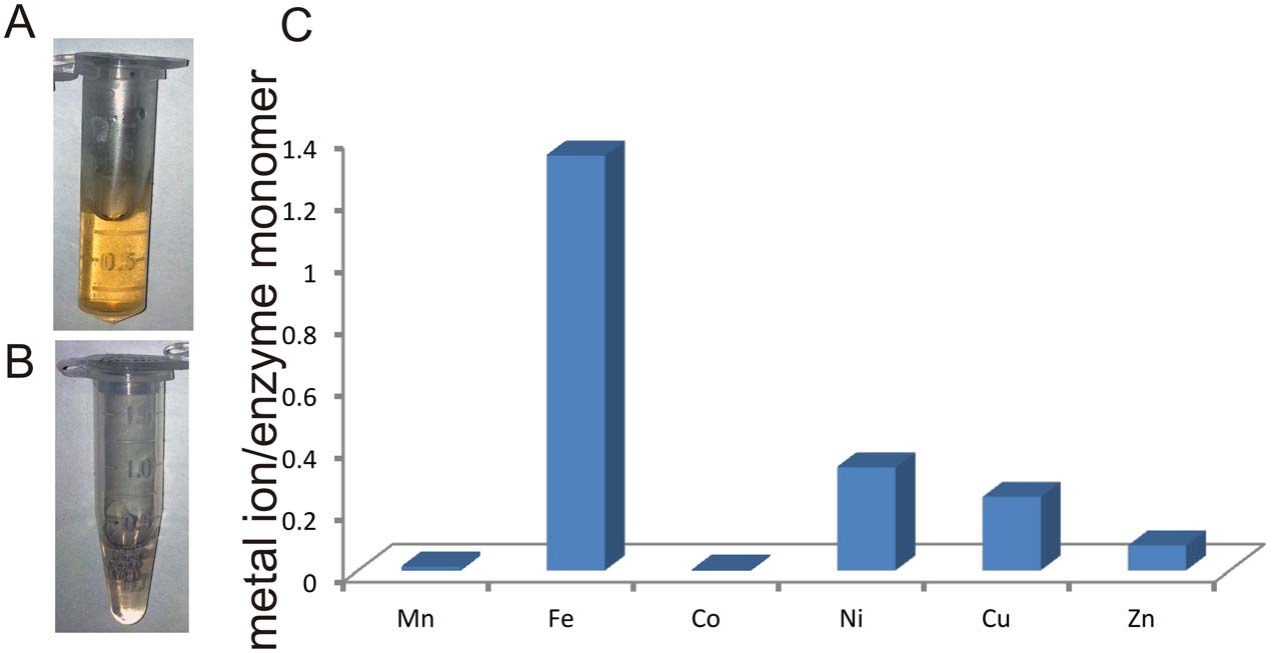
*Rd*DddP contains metal ions. (**a**) Purified *Rd*DddP enzyme at a concentration of ∼10 µM. (**b**) *Rd*DddP after removal of metal ions with chelex resin. (**c**) Plot of the abundance of transition metals in *Rd*DddP identified by ICP-MS.

We cloned, expressed and purified the recombinant putative DddP DMSP lyase (YP_682809) from *R. denitrificans* Och 114 (*Rd*DddP) [12]_,_ [11]. The protein could be prepared to high purity and the concentrated protein solution displayed an amber colour (Figure 2a). When metal chelating chelex resin was added directly to the protein solution the amber coloration disappeared (Figure 2b). This initial result suggested a protein metal complex. To test the hypothesis that *Rd*DddP is a metalloprotein the presence of metal ions in this protein was investigated using inductively coupled plasma mass spectrometry (ICP-MS) and total reflection X-ray fluorescence (TRXF). ICP-MS identified Fe, Ni, Cu, and Zn with occupancies of 1.3, 0.3, 0.2 and 0.1, respectively, per monomer (Figure 2c). TRXF detected the same metals in ratios of 1:0.22:0.14:0.06, for Fe, Ni, Cu, and Zn, respectively (Figure S1 and Table S1 in File S1), which is similar to the results obtained by ICP-MS. These metal ions were measured after extensive dialysis against a buffer from which metals were removed with metal chelating resin. The metal concentrations in this buffer, which served as background control, were below the detection limits of both methods. Combined, these results suggested that the measured metal ions were tightly bound to the protein and that iron constituted the most abundant metal ion in *Rd*DddP.

### Crystal structure of *Rd*DddP revealed a metalloproteinase like fold

**Figure 3:**
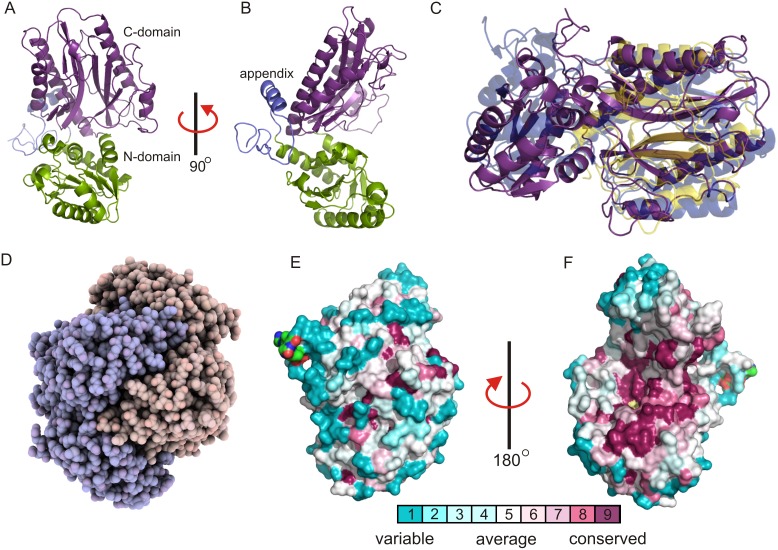
*Rd*DddP has a two-domain structure with the pita bread domain of metallopetidases. (**a**) Frontal view of the pita bread C-domain (purple), the N-terminal domain (green) and the appendix (blue). (**b**) Side view of the enzyme. (**c**): Structural alignment of *Rd*DddP (purple) with the creatinase 1CHM (transparent blue) and the methionine aminopeptidase 1 MAT (transparent yellow). (**d**) Homodimer of *Rd*DddP shown as a sphere model with the two chains in beige and blue. (**e**), (**f**) Surface representations of the monomer with a color code, calculated from multiple DddP lyases revealing that the dimer interface is highly conserved. (**e**) View of the solvent exposed part of the monomer shows little conservation. (**f**) View of the buried dimer interface area shows high conservation. The conservation scores were calculated with the Consurf server using default settings. Structure figures were prepared with Pymol and QuteMol.

To further analyze the putative metalloprotein character of *Rd*DddP we used X-ray crystallography to determine the structure of this enzyme. The X-ray crystal structure of *Rd*DddP was solved using the single-wavelength anomalous dispersion method with a wavelength optimized for crystals of selenomethionine derivatized *Rd*DddP. The preliminary structure determined by this approach served as a search model for molecular replacement using a higher resolution 2.15 Å native dataset (Table S2 in File S1). The crystal structure revealed a two-domain architecture for the 453 residues of *Rd*DddP (molecular weight of 51 kDa) comprising an N-terminal domain of mixed α/β secondary structure and a C-terminal β-barrel domain (Figure 3a, b). Together, the two domains, which are positioned at an angle of about 90° relative to each other, create an elongated shape.

The smaller N-terminal domain (residues 51–206) consists of six β-strands numbered from the N- to the C-terminus (β1, β2, β3, β4, β5 and β6) forming a central β-sheet. In this β-sheet only one β-strand (β3) is anti-parallel. The central β-sheet within the fold is surrounded on both sides by two α-helix bundles consisting of two (α2 and α3) and five α-helices (α4, α5, α6, α7 and α8). Appended to this N-terminal domain is a small N-terminal loop-helix extension (residues 10–50).

At the C-terminus of the protein, the larger β-barrel domain (residues 209–444) is connected through a short hinge region (residues 207–208) to the N-terminal domain. This β-barrel is divided along its longitudinal axis resulting in an open half-β-barrel. The half-β-barrel consists of six anti-parallel β-strands (β7, β8, β9, β10, β11, and β12) of which the first two, β7 and β8, are discontinuous due to a central β-bulge. Aligned to the β-strands, at the exterior wall of the half- β-barrel, are four α-helices (α9, α10, α11, and α12). This C-terminal β-barrel domain, which has been named the “pita bread” domain [14], represents the catalytic domain of the M24B-metallopeptidase family [15], [13].

To identify closely related structural homologs of *Rd*DddP we queried the Protein Data Bank (PDB) using the Dali server [16]. We identified the proline dipeptidase (AAPPs) from *Bacillus anthracis* as closest structural relative (z-score: 32.8, root-mean square deviation of 2.9 Å, and 21% amino acid sequence identity over 343 matched residues; PDB-id: 3Q6D). The structural homolog with the highest pairwise sequence identity was the X-pro aminopeptidase (prolidase) from *Thermotoga maritima* (z-score of 32.26, root-mean square deviation of 2.9 Å, and 23% amino acid sequence identity over 341 matched residues; PDB-id: 2ZSG). These structural homologs, albeit lacking the N-terminal appendix, share the same global organization as *Rd*DddP consisting of two domains. In contrast, the related methionine-aminopeptidases MMAPs (PDB id: 1MAT) contain only the pita bread domain (Figure 3c). Structurally, therefore, the DddP enzymes belong to the metallopeptidase family harbouring the signature pita bread domain.

### *Rd*DddP is a homodimeric enzyme

The crystal structure of *Rd*DddP contained only one molecule per asymmetric unit; however, a tight interaction with a second peptide chain that was related by crystallographic symmetry in the P6_3_22 space group was present, revealing the homodimeric organization of the enzyme. The interaction between the two peptide chains, which is reminiscent of embracing hands (Figure 3d), has been previously described for the creatinase of *P. putida* [14] and prolidases. In *Rd*DddP, this mode of interaction results in a large buried interface area of 3741 Å^2^ and creates a globular tertiary structure with a total surface area of 17701 Å^2^ (Figure 3d). The interaction is mediated by a large array of ionic and hydrogen bond interactions (Table S3 in File S1), calculated with the PDBePISA program [17], [18].

Residues under high positive selection are usually found in the core (folding determinants) or in areas that are important for protein function such as active sites and molecular interfaces [19]. For example, solvent exposed residues located on the surface of proteins are usually less conserved, except if they interact with binding partners. Thus, patches of residues under strong positive selection, i.e. on the surface of a protein, can reveal important biological functions. To calculate the conservation of protein residues we used the Consurf server and mapped the conservation scores obtained from multiple DddP amino acid sequences onto the 3D structure [20]. At the interface of the *Rd*DddP dimer structure we observed a large number of conserved residues almost covering the entire area that is buried in the dimer (Figure 3e, f). This level of conservation at the dimer interface suggested that dimerization is generally present in homologs of *Rd*DddP. Moreover, the homologue *Rn*DddP has been shown to behave as a homodimer with a measured molecular weight of 95.3 in solution [11] comparable to the value of 102 kDa for the crystallographic dimer of *Rd*DddP. Together these results suggested that dimerization is a conserved feature throughout DddP lyases.

### The substrate binding site of *Rd*DddP is shaped by dimerization and hosts a binuclear metal center

**Figure 4:**
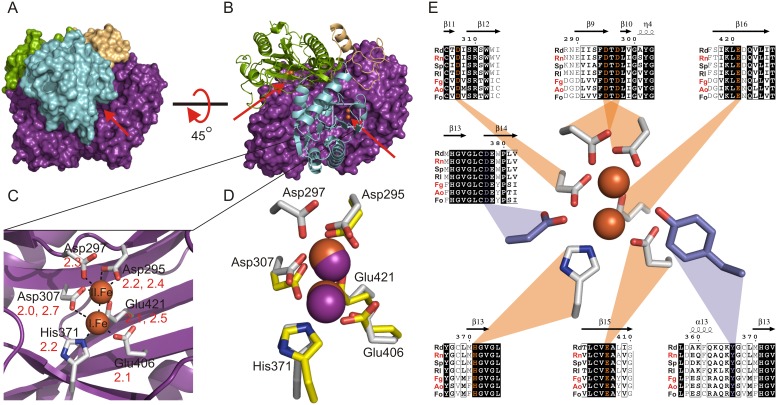
Dimerization shapes the tunnel active sites of *Rd*DddP with a binuclear iron metal center that is conserved in DddP lyases. (**a**) The dimer with one monomer in a cartoon plot presentation (beige, blue, green from the N-to the C-terminus), the second monomer in purple and the iron ions colored in orange. (**b**) The entrance to the two active sites and their path is indicated by the red arrows. (**c**) The metal binding site of *Rd*DddP with the two iron ions in orange and the conserved metal coordinating residues in grey. The distances between residues and metal ions, in angstroms, are indicated in red. (**d**) Comparison of the *Rd*DddP metal binding site (grey, iron ions as orange spheres) with the metal binding site of the methionine aminopeptidase (1MAT), (yellow sticks, cobalt ions as purple spheres). (e) Conservation of metal binding site and the putative catalytic residues in diverse DddP lyases from marine bacteria and fungi. Rd: *Roseobacter denitrificans* OCh 114, Rn: *Roseovarius nubinhibens* ISM, Sp: *Silicibacter pomeroyi* DSS-3, Rl: *Roseobacter litoralis* Och 149, Fg: *Fusarium graminearum* cc19, Ao: *Aspergillus oryzae* RIB40. Fc: *Fusarium culmorum*. Abbreviations in red indicate functionally characterized DddP lyases based on Todd *et al*. 2009. (notably here only a subset of DddP lyases is shown and the residues for metal binding and proposed catalysis are invariant in all DddP lyases presented in Todd *et al*. 2009). The alignment was prepared with ClustalW.

We observed two substrate binding sites per homodimer, which were characterized as pronounced tunnels in the surface of the homodimer structure (Figure 4a, b). Narrow openings of ∼9–11 Å width are located at the dimer interface and form entrances to the active sites. These tunnels are formed by interaction of the N-terminal domains with the pita bread domain of the other peptide chain in the dimer. The N-terminal domain of one chain snugs into the open pita bread fold of the other chain and thereby forms the walls of the tunnels, which end at the active sites located in the center of the pita bread domain [15], (Figure 4b). Hydrophobic residues predominantly line the tunnels and at the base of the tunnel, nested in the center of the pita bread fold, was electron density consistent with a pair of metal atoms (Figure S2 in File S1), which presumably mark the active site of the enzyme (Figure 4c). Due to the absence of strong anomalous signals other than the binuclear metal center in the difference maps of the crystal structure, we can attribute the ions identified by ICP-MS and TRXF as the metals in the active site. Based on the sum of the ion occupancies of 1.9 determined by TXRF, we derived a stoichiometry of about two ions per monomer chain, which supports the binuclear character of the metal center present in the crystal structure. As iron was the most abundant ion that was identified in *Rd*DddP by ICP-MS and TRXF these atoms were modelled as iron in the crystal structure.

Six residues bind these two metal ions: the monodentate Asp297, Glu406, His371, Asp307 and the bidentate Asp295, Glu421 ligands. The distances between these residues and the ions are between 2.1–2.7 Å for metal I, 2.0–2.4 for metal II, and 2.7 Å between the two ions (Figure 4C). Additionally, the B-factor values did not significantly differ between the ions and the ligating atoms. Thus, the properties of these metal-binding sites, when refined as occupied by iron, are consistent with this atom identity. Further supporting this, the interactions between the two ions and protein residues are structurally conserved with the metal binding site of MMAPs (PDB-id: 1MAT), [15], (Figure 4d) where iron ions have been recently identified as cofactor *in vivo*. A residue that is not conserved with MMAPs is Asp297, which may modulate the redox state of the bound metal ion. In conclusion, one monomer provides all the residues that coordinate the metal ions, and while the second dimer approaches the site it does not directly interact with the metal ions, which leaves an open space above them for substrate or water to bind and complete the coordination sphere. Due to the absence of a bound water molecule, product or substrate ligand, the exact geometry of the metal binding site and its complete coordination sphere remains to be established.

Because iron was the most abundant ion present in *Rd*Dddp, as measured by ICP-MS and TXRF, and was identified as the physiological cofactor in the related MMAPs [21] we modeled both metal sites as being occupied by this atom; however, Ni, Zn, and Cu were also identified as cofactors for *Rd*DddP albeit at lower levels. Thus, it is possible that while one metal site is fully occupied by iron, the second site is occupied by a mixture of metal atoms. Indeed, the presence of a stronger and a weaker metal binding site is common in structurally related metallopeptidases and seems to modulate their catalytic activity by alternating metals in the low affinity site [22]. This mixed occupation could also explain the relatively short distance of 2.7 Å between the two modelled ions, which is usually longer (3–3.2 Å) in binuclear iron centers of enzymes [23]. However, such a short distance has been previously reported. For example, the two iron ions in the iron hydrogenase of *Desulfovibrio desulfuricans* are only 2.6 Å apart [24]. It should also be noted that in *Rd*DddP the metal ligands appear to be tightly constrained by the first and second order enzyme residues, leaving not much space between them. Finally, the metal binding residues are invariant in DddP lyases, most likely due to their anticipated function during catalysis (Figure 4e; Alignment S1 in File S1), [12]. Support for this argument is provided by the observation that alanine mutagenesis of any of the metal binding residues in *Rn*DddP, which over all has 77% amino acid identity to *Rd*DddP, led to an inactive enzyme [11].

## Discussion

Based on the structure of the cupin-like DddQ DMSP lyase from *Ruegeria lacuscaelulensis,* the cleavage of DMSP by the DddQ enzymes is proposed to proceed through a β-elimination reaction whereby a bound Zn^2+^ ion coordinates the carboxylate group of DMSP and a tyrosine residue acts as a base to abstract the Cα-H proton and initiate the reaction [25]. The structure of *Rd*DddP suggests a similar reaction mechanism is possible in the DddP family. The strong electropositive charge of the binuclear metal center may attract the negatively charged carboxyl group of the DMSP molecules to bind. This interaction would expose the carbon backbone of DMSP to the two residues, Tyr366 and Asp377, which protrude into the substrate-binding tunnel 4.6–5.5 Å above the metal ions, and both of which are invariant throughout DddP lyases [12], (Figure 4e). Given the common propensity for amino acid side chain carboxylate groups to act as general bases it is likely that Asp337 acts as a base to abstract the Cα-H proton. Given the proposed role of a tyrosine side chain in its phenolate form as a base in the DddQ enzymes, Tyr366 is an alternate candidate for a base in *Rd*DddP. However, unlike in DddQ where the tyrosine ηO atom is involved in coordinating the Zn^2+^ ion and thus maintained in its deprotonated state, such an interaction is not observed for Tyr366 in *Rd*DddP. In this case, it is possible that a proton shuttle involving an adjacent water molecule (HOH 2151) and Asp295, which coordinates one of the iron atoms, enhances the ability of Tyr366 to act as a base. Though, in the absence of additional evidence, we presently favour Asp377 as the most likely candidate to act as the catalytic base. The suggested relevance of metal cofactors for catalysis may also explain the low apparent activity that has been previously measured for *Rn*DddP (Km = 13.8 mM, and Vmax = 0. 31 nmol product/min/µg DMSP), where the low activity may have been a result of measurements made with apo-enzyme or with incorrect metal ions in the active site. While the results presented here suggest metal ions are important for DddP mediated DMSP lysis additional biochemical and kinetic experiments are clearly needed to test this hypothesis.

The presence of iron ions in *Rd*DddP, and the invariance of the metal binding residues in orthologous DddP lyases suggests they are metalloenzymes. Microbial uptake and transport of trace metals to the deep sea, in form of sinking particulates, depletes trace metals in ocean surface waters [28], [29]. Thus, Mn, Co, Cu, Zn and especially Fe can become limiting factors for diverse biological processes. For example low levels of iron limit nitrogen fixation as iron is the cofactor of nitrogenase [30]_,_ [31]. Moreover, iron is a cofactor for photosynthesis and growth of phytoplankton is closely tied to iron concentrations [32], [33], [34]. Thus, the presence of iron in *Rd*DddP raises the question if DddP lyases and DMSP cycling are iron dependent.

Cofactor competition may explain the functional redundancy of different *ddd* genes present in microbial genomes and metagenome datasets. Aside from the DddPs the most abundant DMSP lyases are cupins belonging to the DddQ family. Cupins are small proteins (∼15–30 kDa) with a beta barrel fold, which, similar to the pita bread of M24 metallopeptidases, has emerged as a highly successful scaffold for many enzymatic activities [26]. Most cupins are metalloproteins with a catalytic metal in the center of the β-barrel and these metal binding sites are present in DddQ enzymes [26], [27]. Indeed a Zn^2+^ ion has been recently identified in the active site of the DddQ enzyme from *Ruegeria lacuscaelulensis* [25], a result that is in line with the hypothesis that families of DMSP lyases use different metal cofactors. This diverse scope for metals in DddQ and DddP families may retain enzymatic activity even when one or the other metal species is scarce in the sea.

## Materials and Methods

### Gene amplification

The *Rd*DddP gene was amplified from *R. denitrificans* ISM genomic DNA with the forward primer CATATGGCTAGCATGAACCGTCATTTCAACGC and the reverse primer GTGTGTCTCGAGCTACTCAACGCCCATCAAGGCC. The PCR products were digested with the restriction enzymes NheI and XhoI for *Rd*DddP. The digested PCR products were purified and ligated with an equally digested and purified pET28 vector. Cloning was carried out in *Escherichia coli* DH5α cells and protein expression in BL21 (DE3) star cells.

### Protein production and purification

Five ml of Luria Bertani medium was inoculated with a single colony of BL21(DE3) cells with pET28-*Rd*DddP and incubated at 37°C overnight. One ml of the preculture inoculum was used per 2L ZYP-5052 expression culture (50 mg/ml kanamycin), [35], which was incubated at 20°C, 200 RPM for 4–5 days. The cells were harvested at 6000 g (4°C) and subjected to chemical lysis as previously described [36]. In short, cells were harvested by centrifugation and resuspended in 25 mL sucrose solution (25% w/v), 50 mM Tris-HCl, pH 8.0. Ten milligrams of lysozyme was added to the suspension and stirring was continued for 10 minutes. 50 mL of a deoxycholate solution containing 1% deoxycholate (w/v), 1% Triton X100 (v/v), 20 mM Tris-HCl, pH 7.5, and 100 mM NaCl was added with continued stirring for 10 minutes. The solution was adjusted to 5 mM MgCl_2_ and 2 mg of DNase was added. Cellular debris was separated by centrifuging of the slurry at 15,000 rpm for 45 minutes at 4°C. The lysate was centrifuged, at 27000 g for 45 min, 4°C to pellet cell debris, and the supernatant was loaded onto an immobilized metal affinity chromatography column (GE healthcare), charged with nickel, at room temperature. Fractions were analyzed using SDS-PAGE, pooled based on purity, and diluted into an equal volume of 20 mM Tris-HCl, pH 7.5, and then adjusted to 2.5 mM CaCl_2_. The N-terminal His-tag was always removed. To cleave the His-tag thrombin protease (2 units) was used and the reaction was carried out over night at 4°C. Before the final purification step the proteins were reapplied to the Ni^2+^-column to selectively bind and remove non-digested protein. The flow through was passed over a UNO-Q column (BioRad) for anionic exchange chromatography and eluted in a gradient up to 100% buffer B (buffer A+1 M NaCl). The protein (≥95% purity) was concentrated in a stirred ultrafiltration unit (Amicon) and absorbance at A_280_ was used to determine the final concentration. The concentrated proteins were centrifuged for 20 min at 12000 g, 4°C and the supernatant was filtered though a 0.22 µM filter. The filtered protein solutions was stored at 4°C or directly used for crystallisation.

### Protein Crystallization

Crystallisation screenings were carried out by the vapour diffusion method in sitting drops and the optimizations in hanging drops. *Rd*DddP was crystallized in 4–4.5 M sodium chloride and 0.1 M Bis-Tris pH 6.5 within 1–4 weeks. For cryo-protection the crystals were soaked step wise (5%) until 30% ethylene glycol in mother liquor was reached and they were flash frozen in liquid nitrogen. Both, native and selenomethionine derivative protein (see below) were crystallized under comparable conditions.

We generated selenomethionine substituted protein using a combination of the Studier autoinduction minimal medium and the selenomethionine nutrient mix (Athena, Enzyme Systems) as previously described [36]. The heavy metal substructure identification, phasing and phase improvement were all carried out with AutoSHARP [37] followed by initial model building in Buccaneer. This preliminary model was used to solve the native structure by molecular replacement [38]. The model was manually completed in Coot [39] and refined against the native data with REFMAC5 [40]. Unless otherwise stated all crystallographic programs were used as part of the CCP4 project [41]. Figures were prepared with Pymol (www.pymol.org) and QuteMol [42]. The crystal structure was verified with Molprobity [43].

### Sample preparation for metal identification

To remove extraneously bound metal ions from the enzymes (200 µl/10–40 mg/ml) of the purified proteins were extensively dialysed (Millipore, 10 kDa cutoff, polyethersulfone) against 20 mM Tris-HCl, pH 7.5, 200 mM NaCl, which was previously demetallized with Chelex resin (BioRad). The buffer (50 ml), which further included 1 g of Chelex resin, was exchanged three times during two days at 4°C. The buffer of the last dialysis step served as a (metal free) control for all subsequent metal analysis procedures.

### Metal identification by inductively coupled plasma mass spectrometry (ICP-MS)

Each MS-spectrum was recorded in duplicate for which 100 µL aliquots of each sample were digested in 1 mL of 16N Nitric Acid (Anachemia Environmental Grade) in clean Teflon digestion vessels. These samples were heated to approximately 120°C overnight until the digestion was complete. The samples were then quantitatively transferred to clean polyethylene sample vials and diluted to 50 mL with 18.2 mega-ohm deionized water. Trace element analysis was performed on a Thermo X-Series II quadrupole ICP-MS run in standard mode with glass concentric nebulizer, Peltier cooled glass impact bead spray chamber, and on-line internal standard addition. A mixture of Rh, In, and Re was used as the internal standard. Calibration standards were prepared from NIST traceable mixed element stock solutions. Instrumental precision and accuracy were determined by six replicate analyses of the Certified Reference Material SLRS-5 (Ottawa River Water, NRCC), these replicates were spread over the course of the analytical run.

### Metal identification by total reflection X-ray fluorescence (TRXF)

100 µl aliquots of each sample were placed onto the center of a standard quartz TXRF carrier and dried within 20 min under an IR lamp. A Bruker S2 Picofox TRXF device equipped with an auto sampler and a Mo-micro X-ray tube was used for trace element investigation at room temperature. By excitation with the Mo(Kα) line (17.4 keV) for 500 s, a multi-element fluorescence spectrum was obtained. A 10 mg/L solution of gallium was applied as an internal standard to quantify the trace elements. The specific Kα lines were used for the determination of the elements investigated. The signal of the most abundant metal was set to 1.0, and all other metals were quantified relative to the most abundant metal.

**For Bioinformatics** such as calculations of conservation scores we used the Consurf server with default settings using *Rd*DddP as search structure [20], [19]. Alignments were created with ClustalW [44] as part of the Bioedit program [45] and the secondary structure information of the *Rd*DddP structure was superposed onto the alignment using ESPript [46].

### Accession Codes

Protein Data Bank: Coordinates and structure factors have been deposited with the following accession number of **4b28**.

## Supporting Information

File S1 **Supplementary Figures S1–S2, Supplementary Tables S1–S3, Supplementary alignment S1.**
(DOCX)
