# Supplementary material for: The Structure of RdDddP from Roseobacter denitrificans Reveals That DMSP Lyases in the DddP-Family Are Metalloenzymes
Source: PLoS One. 2014 Jul 23;9(7):e103128. doi: 10.1371/journal.pone.0103128 (PMC4108388; doi:10.1371/journal.pone.0103128)
Supplement: File S1 — Supplementary Figures S1–S2, Supplementary Tables S1–S3, Supplementary alignment S1. (DOCX) [file pone.0103128.s001.docx]

**Supporting Information:**

The structure of *Rd*DddP from *Roseobacter denitrificans* reveals that DMSP lyases in the DddP-family are metalloenzymes

Jan-Hendrik Hehemann^1,3^, Adrienne Law^1^, Lars Redecke^2^ & Alisdair B. Boraston^1^

^1^Department of Biochemistry & Microbiology, University of Victoria, PO Box 3055 STN CSC, Victoria, British Columbia, V8W 3P6, Canada

^2^Joint Laboratory for Structural Biology of Infection and Inflammation of the Universities of Hamburg and Lübeck, c/o DESY, 22603 Hamburg, Germany

^3^ Current address: Department of Civil and Environmental Engineering, Massachusetts Institute of Technology, 15 Vassar Street, Bldg 48-108, Cambridge, USA MA 02139

*Correspondence should be addressed to: Jan-Hendrik Hehemann ([jh.hehemann@gmail.com](mailto:jh.hehemann@gmail.com)) or Alisdair B. Boraston (boraston@uvic.ca)

**Supporting Information Legend**

Supplementary Figures S1-S2

Supplementary Tables S1-S3

Supplementary Alignment S1

**
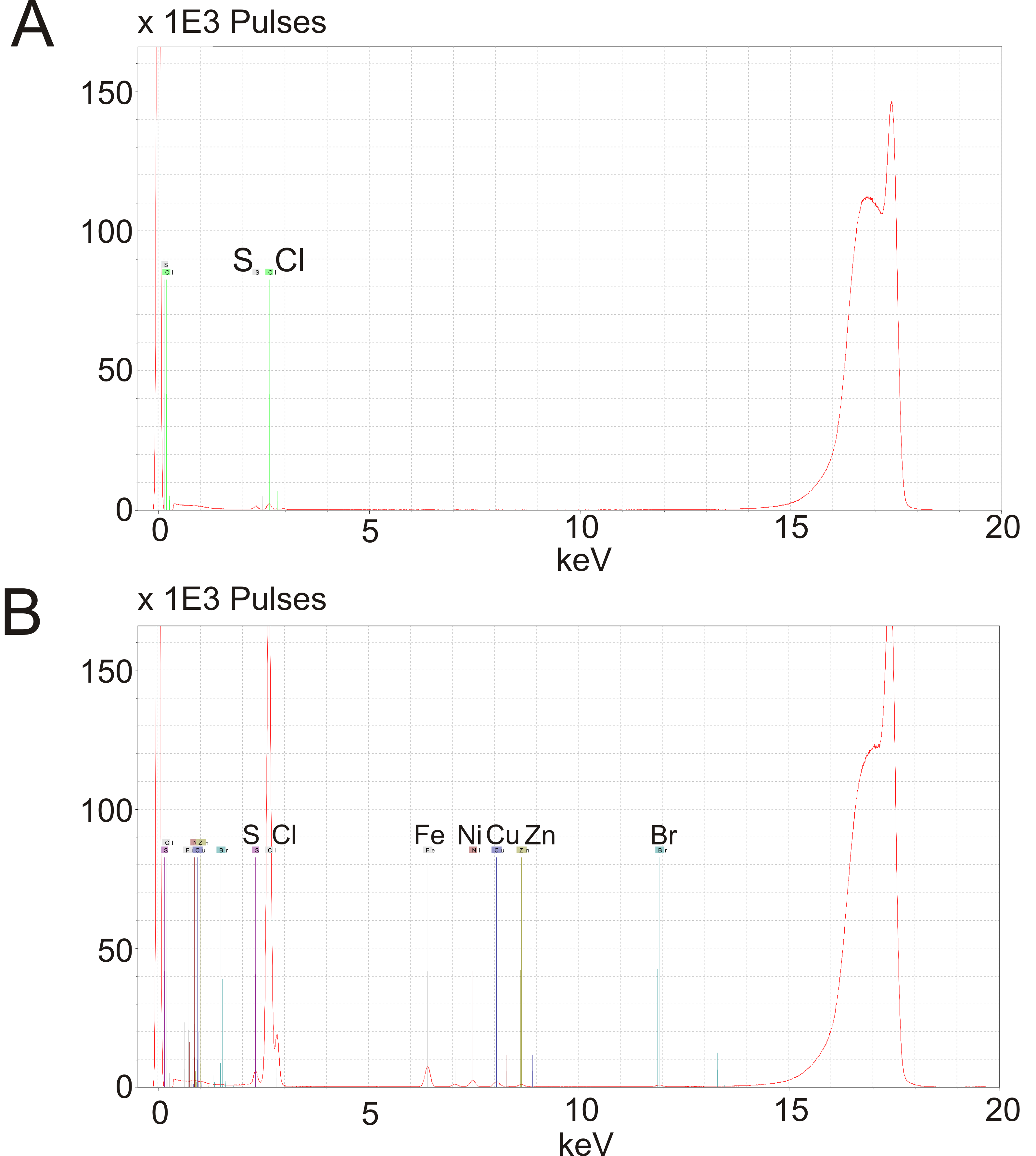
**

Figure S2: Metal analysis of *Rd*DddP by total reflection X-ray fluorescence (TRXF) revealed iron as most abundant cofactor. A) X-ray fluorescence spectrum of the metal free buffer against which *Rd*DddP was dialyzed. B) X-ray fluorescence spectrum of *Rd*DddP measured in the metal free buffer.

**Table S1: Metal analysis of *Rd*DddP by total reflection X-ray fluorescence (TRXF).**

| **No.** | **Element** | **Line** | **Energy/keV** | **Cycl.** | **Net** | **Backgr.** | **Conc./(g/l)** | **Conc./(mmol/l)** |
| --- | --- | --- | --- | --- | --- | --- | --- | --- |
| 1 | Cl | K12 | 2,622 | 40 | 4592899 | 48515 | 305,72 |  |
| 3 | Fe | K12 | 6,405 | 40 | 226364 | 23940 | 1 | 17,09 |
| 4 | Ni | K12 | 7,48 | 40 | 75637 | 24333 | 0,23 | 3,92 |
| 5 | Cu | K12 | 8,046 | 40 | 60657 | 24469 | 0,164 | 2,58 |
| 6 | Zn | K12 | 8,637 | 40 | 28217 | 24707 | 0,066 | 1,01 |
| 9 | Br | K12 | 11,924 | 40 | 20677 | 34118 | 0,03 | 0,38 |
| 10 | Br | L1 | 1,481 | 40 | 550 | 44587 |  |  |
| 14 | Mo | K12 | 17,48 | 40 | 25103904 | 817368 |  |  |
| 15 | Mo | L1 | 2,292 | 40 | 125323 | 51888 | 150,007 |  |

Figure S2: The metal binding site of *Rd*DddP. The electron density of the binuclear metal site is shown in mesh (prior to modelling the atoms) as an unbiased maximum likelihood/σ_a_-weighted F_o_-F_c_ map (blue) contoured at 3σ (0.15e/Å^3^) and an anomalous difference map (green) at 3σ (0.03e/Å^3^) calculated with data collected at a wavelength of 1.3379 Å. The modeled Fe atoms are shown as brown spheres and coordinating residues as grey sticks.

**Table S2: Data collection and refinement statistics.**

|  | **RdDddP_Semet** | **RdDddP_native** |
| --- | --- | --- |
| **Data collection** |  |  |
| Beam Line | CLS | CLS |
| Wavelength (Å) | 0.9788 | 1.3379 |
| Space group | P6_5_22 | P6_3_22 |
| **Cell dimensions (Å)** |  |  |
| *a*, *b*, *c*, (Å) | 117.7, 117.7, 405.9 | 116.9, 116.9, 133.5 |
| α, β, γ (°) | 90, 90, 120 | 90, 90, 120 |
| Resolution (Å) | 80-2.8 (2.96-2.8) | 50-2.15 (6.80-2.15) |
| R_merge_ | 0.115 (0.32) | 0.12 (0.40) |
| *I*/σ*I* | 21.1 (9.8) | 15.3 (3.4) |
| Completeness (%) | 99.9 (99.4) | 98.9 (93.6) |
| Redundancy | 15.4 (12.5) | 13.6 (4.2) |
| **Refinement** |  |  |
| Resolution (Å) |  | 2.15 |
| No. reflections |  | 27928 |
| R_work_ / R_free_ |  | 0.21/0.25 |
| **No. atoms** |  |  |
| Protein |  | 3394 |
| Ligand^b^ |  | N.D. |
| Water |  | 201 |
| **B-factors** |  |  |
| Protein |  | 28.4 |
| Water |  | 29.3 |
| **R.m.s. deviations** |  |  |
| Bond lengths (Å) |  | 0.014 |
| Bond angles (°) |  | 1.3 |
| **Ramachandran statistics (%)** |  |  |
| Favored |  | 96.8 |
| Allowed |  | 3.0 |
| Disallowed |  | 0.2 |

Values in parentheses are for the highest-resolution shell.

**Table S3. *Rd*DddP homodimer interface table showing contact distances within the homodimer interface that indicate the presence of hydrogen bonds and ionic interactions (top part of table) and potential interactions (bottom half).**

| [Structure 1](javascript:openWindow('pi_ipage_atom1.html',400,250);) | [Dist. [Å]](javascript:openWindow('pi_ipage_atmdist.html',400,250);) | [Structure 2](javascript:openWindow('pi_ipage_atom2.html',400,250);) |
| --- | --- | --- |
| A:ARG 273[ NE ] | 2.86 | A:ARG  29[ O  ] |
| A:ARG 273[ NH2] | 2.87 | A:VAL  30[ O  ] |
| A:LYS 177[ NZ ] | 2.86 | A:HIS  98[ O  ] |
| A:TYR 395[ OH ] | 3.65 | A:TYR 141[ OH ] |
| A:TYR 393[ OH ] | 2.69 | A:PHE 142[ O  ] |
| A:TYR 395[ OH ] | 2.52 | A:ASP 146[ OD2] |
| A:ARG 257[ NE ] | 3.11 | A:ASP 246[ OD1] |
| A:ARG 266[ NH1] | 3.06 | A:GLY 260[ O  ] |
| A:TRP  95[ N  ] | 2.93 | A:GLU 264[ O  ] |
| A:HIS  98[ NE2] | 2.68 | A:GLU 264[ OE1] |
| A:THR  90[ OG1] | 2.48 | A:GLU 280[ OE1] |
| A:MET  92[ N  ] | 3.23 | A:GLU 280[ OE2] |
| A:ASN 119[ ND2] | 3.88 | A:LYS 365[ O  ] |
| A:ASP 146[ N  ] | 2.97 | A:ALA 392[ O  ] |
| A:ARG 144[ N  ] | 3.14 | A:TYR 393[ OH ] |
| A:GLY 145[ N  ] | 3.28 | A:TYR 393[ OH ] |
| A:TYR 141[ OH ] | 3.65 | A:TYR 395[ OH ] |
| A:ARG  29[ O  ] | 2.86 | A:ARG 273[ NE ] |
| A:VAL  30[ O  ] | 2.87 | A:ARG 273[ NH2] |
| A:HIS  98[ O  ] | 2.86 | A:LYS 177[ NZ ] |
| A:PHE 142[ O  ] | 2.69 | A:TYR 393[ OH ] |
| A:ASP 146[ OD2] | 2.52 | A:TYR 395[ OH ] |
| A:ASP 246[ OD1] | 3.11 | A:ARG 257[ NE ] |
| A:GLY 260[ O  ] | 3.06 | A:ARG 266[ NH1] |
| A:GLU 264[ O  ] | 2.93 | A:TRP  95[ N  ] |
| A:GLU 264[ OE1] | 2.68 | A:HIS  98[ NE2] |
| A:GLU 280[ OE1] | 2.48 | A:THR  90[ OG1] |
| A:GLU 280[ OE2] | 3.23 | A:MET  92[ N  ] |
| A:LYS 365[ O  ] | 3.88 | A:ASN 119[ ND2] |
| A:ALA 392[ O  ] | 2.97 | A:ASP 146[ N  ] |
| A:TYR 393[ OH ] | 3.14 | A:ARG 144[ N  ] |
| A:TYR 393[ OH ] | 3.28 | A:GLY 145[ N  ] |
| A:ARG 257[ NH2] | 3.93 | A:ASP 246[ OD1] |
| A:ARG 257[ NE ] | 3.11 | A:ASP 246[ OD1] |
| A:HIS  98[ NE2] | 2.68 | A:GLU 264[ OE1] |
| A:HIS  98[ NE2] | 3.44 | A:GLU 264[ OE2] |
| A:ASP 246[ OD1] | 3.11 | A:ARG 257[ NE ] |
| A:ASP 246[ OD1] | 3.93 | A:ARG 257[ NH2] |
| A:GLU 264[ OE1] | 2.68 | A:HIS  98[ NE2] |
| A:GLU 264[ OE2] | 3.44 | A:HIS  98[ NE2] |

**Supplementary alignment S1**

**>Roseobacter_denitrificans_OCh_114{110679802}**

**RGATLGDGSPNDMNRVEIGPTQLAFAEWHTARLDLPDLAAMRRFRHRRLTDHVVARGYAG**

**LLMFDPLNIRYATDSTNMQLWNTHNPFRATLLCADGYMVMWDYKNSPFLSEFNPLVREQR**

**AGADLFYFDRGDKVDVAADVFANEVRILLRD-HAPGLRRLAVDKVMLHGLRALQAQGFEI**

**MDGEEVTEKARSVKGPDEIRAMRCASHACEVAVRKMEDFARSKVGDGVTCENDIWAILHS**

**ENVRRGGEWIETRLLASGPRSNPWFQECGPRVCQRNEIISFDTDLVGAYGICTDISRSWW**

**I-GDQKPRADMIYAMQHGVEHIRTNMEMLKPGVMIPELSANTHVL-DAKFQKQKYGCLMH**

**GVGLCDEWPLVAYP-DHAVAGAYDYPLEPGMTLCVEALISEEGGDFSIKLEDQVLITEDG**

**-YENLTKYPFDPALMGVE**

**>Roseovarius_nubinhibens_ISM{83950505}**

**RGATLGDNTPNDNNRIEIGPTQLAFGEWATAGLALPDLQRMREFRWNRLTQAVVDRDYGG**

**VLMFDPLNIRYATDSTNMQLWNAHNPFRALLVCADGYMVIWDYKNSPFLSTFNPLVREQR**

**FGADLFYFDRGDKVDVAADAFSNEVRTLIAE-HGGGNMRLAVDKIMLHGLRALEAQGFEI**

**MEGEELTEKTRAIKGPDEILAMRCAVHACETSVAAMEHFAREAVPQGNTSEDDVWAVLHA**

**ENIKRGGEWIETRLLASGPRTNPWFQECGPRIIQNNEIISFDTDLIGSYGICVDISRSWW**

**V-GDAAPPADMVYAMQHAHEHIMTNMEMLKPGVTIPELSERSHRL-DEQFQAQKYGCLMH**

**GVGLCDEWPLVAYP-DQAVPGSYDYPLEPGMVLCVEAAVGAVGGNFTIKLEDQVLITETG**

**-YENLTSYPFDPALMGR-**

**>Silicibacter_pomeroyi_DSS-3{56697158}**

**----------------------------------------MREHRWKRLTRAIVDRGYGG**

**LLMFDPLNIRYATDSTNMQLWNTHNPFRAVLLCADGYMVIWDYKNSPFLSTFNPLVREQR**

**SGADLFYFDRGDKVDVAADVFSNEVRVLIEA-HSGGNRRLAVDKIMLHGLRALEAQGFEI**

**MEGEEVTEKTRAIKGPDEILAMRCAHHACESAIAEMERFARANVGDGKTTEDDIWAVLHA**

**ENIRRGGEWIETRLLASGQRTNPWFQECGPRITQKNEVIAFDTDLIGSYGICIDISRSWW**

**I-GDQAPRPDMIYAMRHAHEHIMTNMEMLKPGVMIPELTANCHRL-DDKYQAQKYGCLMH**

**GVGLCDEWPLVAYP-DKAVAGAYDYALEPGMVLCVEACVGEVGGDFSIKLEDQVLITEDG**

**-YENLTRYPFDPALMGQA**

**>Roseobacter_litoralis_Och_149{339503515}**

**RGATLGDGSPNDMDRVEIGPTQLAFAEWQEAGLELPDLAAMRRFRHERLTAHIVARGYAG**

**LLMFDPLNIRYATDSTNMQLWNTHNPFRATLLCADGYMVMWDYKNSPFLSEFNPLVNEQR**

**SGADLFYFDRGDKVDVAADVFANEVRVLLRN-HAPELRRLAVDKVMLHGLRALQAQGFEI**

**MDGEEVTEKARSVKGPDEIRAMRCASHACEVAVRKMEDFARSNVGDGVTCENDIWAILHS**

**ENVRRGGEWIETRLLASGPRSNPWFQECGPRICQMNEIISFDTDLVGVYGICTDISRSWW**

**I-GDQKPRADMIYAMQHGVEHIQTNMEMLKPGVMIPELSANTHVL-DAKFQKLKYGCLMH**

**GVGLCDEWPLVAYP-DHAVEGAYDYPLEPGMTLCVEALVSEEGADFSIKLEDQVLITEDG**

**-YENLTQYPFDPALMGTT**

**>Fusarium_graminearum_cc19{193527459}**

**HATSKPDGSDNNNDRVEIGPTPLAFREWRALGLQPPHLPTMRAYRLQRIRDELTNRDLGG**

**ILLFDPLNIRYATDTTNMQLWTTHNPARACFVAASGYVVLWDFHGCNHLSAHLPLINERR**

**SGASFFYFETGNRTDEQAARFATQIDQLLRQ-HAGNNRRLAVDRIEVAGLRALDALGLDV**

**CNGQVVTEHARMIKGPDEILAMRCAVASCEAAIGEMRQVMRA-----GATENDVWAALHA**

**GNIRRGGEWIETRLLSSGPRTNPWYQECGPRVLRDGDLVVFDTDLIGVYGICVDVSRTWI**

**C-GDLEPTAEQKRLYRIAHEHITTNIEMVKPGVRFTDLTRNGHRL-PESCRAQRYGVMFH**

**GVGLCDEYPSIRYP-EDLESYGYEGEIQAGMVLCVEAYVGEVGGKDGVKLENQLLVTDTG**

**-YELLTHYPFEQSFLD--**

**>Aspergillus_oryzae_RIB40{169778893}**

**TATFKPDGSDNDNDRVEVGPTPLAFAEWQHLGLQPPHLPTMRAYRLQRICDQLISRDLGG**

**ILLFDPLNIRYATDTSNMQLWTAHNPSRACFVAASGYLVLWDFHGCNHLSAHLPLIKETR**

**SGASFFYFETGNRTDEHAARFCAQVDELLRK-HAGNNRRLAVDRIEVAGLRALDALGVEV**

**CNGQAVTELARMIKGPDEIRAMRCAVASCEAAVGEMRQAMRA-----GATENDVWAALHA**

**GNIRRGGEWIETRLLSSGPRTNPWYQECGPRILRDGDLVSFDTDLIGVYGICVDMSRSWI**

**C-GDLEPTAEQKRLYRIAHEHITNNIEMVKPGVRFTELTRNGHRL-PESCRAQRYSVMFH**

**GVGLCDEYPTIRYP-EDLESYGYEGELQAGMVLCVEAYVGEVGGKDGIKLENQLLVTETG**

**-YELLTRYPFEESFLRD-**

**>Fusarium_culmorum{193527461}**

**IATSKPDGSDNNNDRVEIGPTPLAFSEWRALGLQPPHLPTMRAYRLQRIRDELANRDLGG**

**ILLFDPLNIRYATDTTNMQLWTTHNPARACFVAASGYVVLWDFHGCNHLSAHLPLINERR**

**SGASFFYFETGNRTDEQAARFATQIDQLLRQ-HAGNNRRLAVDRIEVAGLRALDALGLDV**

**CNGQVVTEHARMIKGPDEILAIRCAVASCEAAIGEMRQAMRA-----GATENDVWAALHA**

**GNIRRGGEWIETRLLSSGPRTNPWYQECGPRVLRDGDLVVFDTDLIGVYGICVDVSRTWI**

**C-GDLEPTAEQKRLYRIAHEHITTNIEMVKPGVRFTDLTRNGHRL-PESCRAQRYGVMFH**

**GVGLCDEYPSIRYP-EDLESYGYEGELQAGMVLCVEAYVGEVGGKDGVKLENQLLVTDTG**

**-YELLTHYPFEQSFLD--**
